# Ultrafast collisional ion heating by electrostatic shocks

**DOI:** 10.1038/ncomms9905

**Published:** 2015-11-13

**Authors:** A. E. Turrell, M. Sherlock, S. J. Rose

**Affiliations:** 1 Blackett Laboratory, Imperial College London, Prince Consort Road, South Kensington, London SW7 2AZ, UK; 2 Clarendon Laboratory, University of Oxford, Oxford OX1 3PU, UK

## Abstract

High-intensity lasers can be used to generate shockwaves, which have found applications in nuclear fusion, proton imaging, cancer therapies and materials science. Collisionless electrostatic shocks are one type of shockwave widely studied for applications involving ion acceleration. Here we show a novel mechanism for collisionless electrostatic shocks to heat small amounts of solid density matter to temperatures of ∼keV in tens of femtoseconds. Unusually, electrons play no direct role in the heating and it is the ions that determine the heating rate. Ions are heated due to an interplay between the electric field of the shock, the local density increase during the passage of the shock and collisions between different species of ion. In simulations, these factors combine to produce rapid, localized heating of the lighter ion species. Although the heated volume is modest, this would be one of the fastest heating mechanisms discovered if demonstrated in the laboratory.

## Abstract (web summary)

Short pulses of high intensity laser light usually heat the ions in dense plasmas indirectly via collisions with the electrons. Here, the authors identify an extremely rapid alternative heating mechanism based on ion-ion collisions.

## Graphical abstract

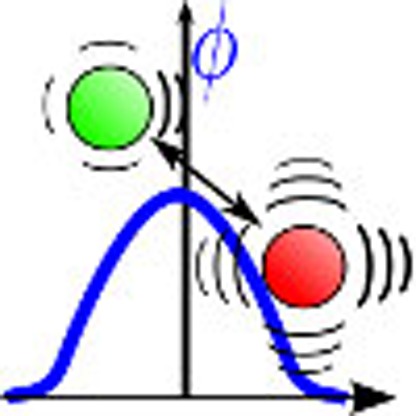

High-intensity (>10^18^ W cm^−2^), high-contrast (10^9^) short-pulse (<100 fs) lasers have allowed for the creation and manipulation of close to solid density plasma. This is because they allow for direct laser–target interaction before hydrodynamic expansion can create a lower density pre-plasma, which absorbs much of the laser pulse^1^,^2^. In these interactions, ions are usually only indirectly heated by the laser via electrons; typically, ions gain thermal energy only through electron–ion thermal equilibration or electron–ion instabilities^3^. There are several mechanisms for the deposition of laser energy into ions such as indirect heating by electrons, ion trapping in the downstream region of shocks or through the acceleration of bunches of ∼10^9^ ions to ∼MeV energies for a broad range of applications including nuclear fusion, proton radiography and hadron therapy^4^,^5^,^6^,^7^,^8^,^9^,^10^,^11^,^12^,^13^,^14^,^15^,^16^,^17^. Dissipation of laser energy through the intermediary of collisionless electrostatic shocks (CESs)^18^,^19^,^20^ are one such class of deposition mechanisms. Ion–ion collision dynamics are often assumed to be unimportant in these cases, which typically involve near-critical density targets. In the solid density targets we consider, the ion–ion dynamics are critical for the deposition of laser energy into ions.

In the following, we demonstrate an entirely unexpected effect; for CESs launched into solid density targets composed of two ion species, the dissipation mechanism of CESs can switch from acceleration of a few ions to bulk heating of the target to keV temperatures. Moreoever, the heating process is extremely rapid, on time scales of femtoseconds, and is unusual because electrons play no direct part in the thermal heating of the ions. The ability to create small regions of very high ion energy density on time scales shorter than that of hydrodynamic expansion will be of interest in attempts to understand the processes involved in inertial confinement fusion^21^ and will have implications for some specialized ion acceleration schemes which involve solid density targets. The effect is unimportant for densities much less than solid including near-critical density targets at typical wavelengths for high-intensity laser systems.

## Results

### Collisionless electrostatic shocks

CESs are dissimilar to the planar plasma shocks typically encountered in high energy density physics. Planar plasma shocks heat material with just a single ion species^22^, they have electric fields that do not change sign over the shock width, *L*, and the usual dissipation mechanism is via ion–ion collisions^23^. These shocks are described by the Rankine–Hugoniot relations^24^.

In contrast, CESs exhibit a bipolar electric field that moves with the shock and have a different theoretical description. Most applications of CESs involve ion acceleration via reflection from the shock front^18^,^20^,^25^,^26^. Reflection from the shock front is the usual dissipation mechanism in near-critical density targets, whereas dissipation through heating is the dominant dissipation mechanism in weak shocks. Dissipation can also occur via ion trapping in the region downstream of the shock.

CESs formation is described by the Sagdeev potential^27^, which admits either shock or soliton solutions when Φ(*ϕ*)<0 for Φ(*ϕ*)=*P*_*i*_(*φ*, *M*)−*P*_*e*1_(*ϕ*, Θ, Γ, *β*_*e*0_)−*P*_*e*0_(*ϕ*, Γ, *β*_*e*0_) with 0 subscripts denoting upstream parameters, 1 downstream parameters, *P* pressure, *M*=*v*_sh_/*C*_s_ the Mach number of the shock, *ϕ*=*eφ*/*T*_*e*0_ the normalized electrostatic energy across the shock front, Γ=*n*_*e*1_/*n*_*e*0_, Θ=*T*_*e*1_/*T*_*e*0_ and *β*_*e*0_=*m*_*e*_*c*^2^/*T*_*e*0_ the normalized inverse electron temperature. High-intensity lasers have laser pressures in excess of the electron pressure on the front surface of the target, 
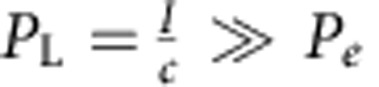
. Front surface electrons are heated to the ponderomotive energy within a half-laser cycle, so that 
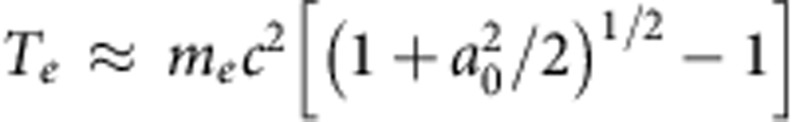
, for normalized laser parameter 
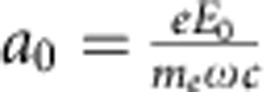
5. This causes a front surface increase in electron pressure such that Φ(*φ*)<0 and a CES can be launched. At very high intensities *P*_L_ remains high and hole boring^28^ of the target can take place. In this study, we focus on the regime in which CESs are launched but no significant hole boring takes place. This corresponds to modest values of the hole-boring parameter Ξ=*I*/(*ρc*^3^) of Ξ=10^−6^–10^−3^. After formation, the shock decouples from the laser pulse and propagates independently through the target until its energy is dissipated. Its initial velocity is similar to the initial hole-boring speed and is given in the non-relativistic case relevant here by 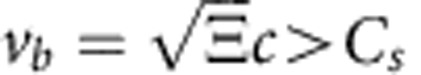 (ref. 29) where *C*_*s*_ is the ion sound speed.

In ion acceleration by CESs, ions approach the shock front with speed −*v*_sh_ in the frame co-moving with the shock and are accelerated to *v*_sh_. In the laboratory frame, the ions reflected from the shock front gain a maximum momentum 
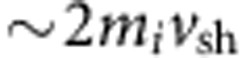
.

### Ultrafast collisional ion heating mechanism

CES dissipation can be through ion acceleration or through heating^27^,^30^. For a single ion species and a strong shock, the passage of a CES does not heat the ions, although reflected ions may become trapped in the downstream region and cause heating ahead of the shock. However, in material composed of two different ion species we find that the CES dissipation mechanism can switch from energy being mostly dissipated via ion acceleration to a significant fraction being dissipated as thermal energy of the lighter ion species during passage of the shock. This is particularly true for high-density material. Although the formation of the shock is not novel here and follows the usual collisionless formation process, the dissipation mechanism becomes collisional and occurs during passage through the shock.

This process occurs because the two ion species are differentially accelerated in proportion to their charge-to-mass ratio by the electric field, *E*_*x*_, of the shock; 
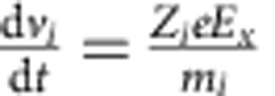
 for ion species *i*. Differential acceleration of two different ion species *i* and *j* causes a non-zero relative velocity *v*_*ij*_=*v*_*i*_−*v*_*j*_ between them. Rapid heating of the ions proceeds via dynamical friction between the two species of ion. The higher temperature of the lighter ion species in the material with two different ion species causes a greater proportion of the lighter ion species to pass through the shock^31^, rather than be reflected, significantly changing the CES energy deposition into the target.

**Figure 1:**
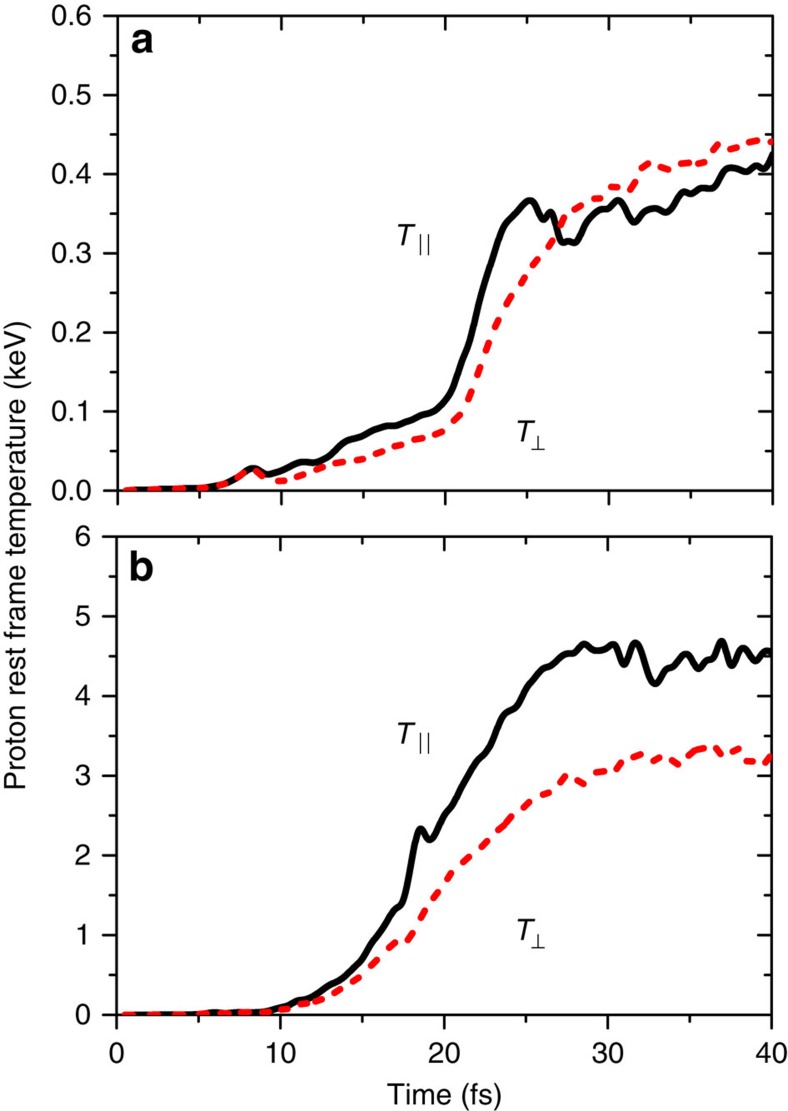
Temperature rise due to ultrafast collisional ion heating as calculated in PIC simulations. Temperatures are shown in the directions parallel, *T*_||_, and perpendicular, *T*_⊥_, to the laser propagation direction and are each an average of the ten computational cells (approximately a skin depth *δ*_sk_) on either side of the highest density point in the target. The temperatures are fitted as demonstrated in Fig. 4. The simulations included collisions between all species, electron and ion. (**a**) CH target, *I*=10^20^ W cm^−2^; (**b**) CsH target, *I*=5 × 10^20^ W cm^−2^.

We have found that the rate of heating is extremely rapid, as shown in Fig. 1 for two different target types. Plastic is a ubiquitous laser target, while caesium hydride (CsH) is chosen as a target because it has properties such as its typical charge and therefore charge-to-mass ratio, which are in particular suited for ultrafast collisional ion heating. The figure is described in more detail in the Simulation results section. The rate of heating of the ions is accounted for by the fact that the ion–ion collisions, which drive this heating mechanism, occur on shorter time scales than the electron–ion interactions that typically cause ion heating in laser–plasma interactions. Usually, laser plasma interactions deposit energy into electrons and ions are heated indirectly through instabilities involving electrons or through electron–ion equilibration (driven by electron–ion collisions). This can be seen by comparing the rate of change of temperature due to the collisional ion heating, equation (3) of the analytical model found in the Methods section, to the Landau–Spitzer electron–ion temperature equilibration rate^32^,^33^. Assuming that the relative ion velocity is *v*_*ij*_≈*v*_th,*ij*_,


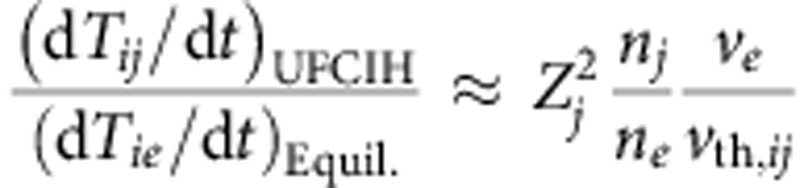


then the ion temperature increase due to ultrafast collisional ion heating can dominate electron–ion equilibration for relativistic laser–plasma conditions. The second reason for the rapid rate of heating is the density increase, which occurs during the passage of a CES. The collisional absorption of energy per unit time scales is


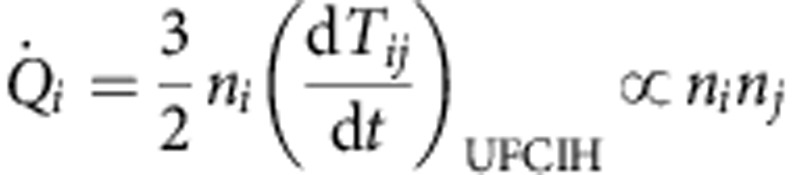


and the shock passage inside the target, where the heating occurs, causes density increases across the shock of ∼2–8 times the initial density.

### Simulation results

The results of simulations, obtained via the process described in the Methods section, are shown in the figures. The figure panels are labelled a–c for plastic (CH) and d–f for CsH. We present three different scenarios; a and d are collisions enabled between all species (electrons and ions), b and e are the same as a and d, respectively, except that inter-ion collisions are disabled (collisions between ions of different species), and c and f are the same as a and d, respectively, except that the second, more massive ion species is not present in the material (a single species equivalent case to the first scenario). These three different scenarios show the effects and mechanism of the ultrafast collisional ion heating clearly. In a and d ultrafast collisional ion heating occurs, whereas in b, c, e and f it cannot, because it relies on collisions between different ion species.

**Figure 2:**
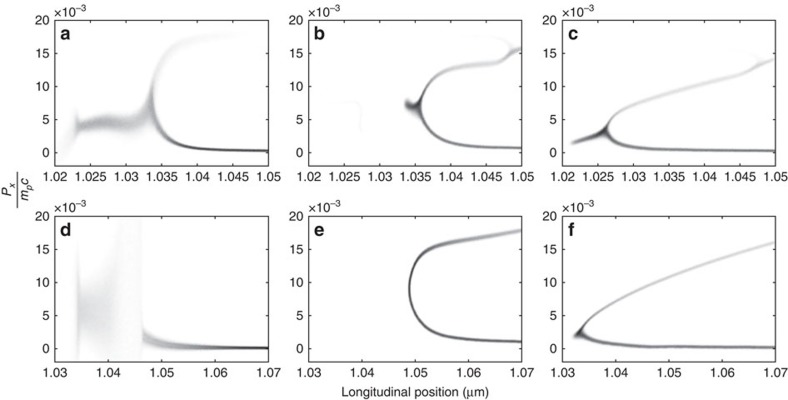
Phase space of protons showing the shock front 20 fs into 40-fs-long PIC simulations. (**a**,**d**) All collisions enabled; (**b**,**e**) collisions between different ion species disabled; (**c**,**f**) all collisions enabled but the material is made up of protons only at the same density as the protons in **a**,**d**, respectively; (**a**–**c**) CH target, *I*=10^20^ W cm^−2^; (**d**–**f**) CsH target, *I*=5 × 10^20^ W cm^−2^. The heating is evident in **a**,**d** as an increase in the spread of *P*_*x*_ upstream of the shock front. The other panels show that without collisions between different species of ion, there is significant reflection of protons from the shock front.

Figure 2 shows the phase space of protons from the three different scenarios for both target types. The spread in *P*_*x*_ is larger and the number of protons reflected far less, in scenario a and d when the thermal dissipation mechanism is activated by the presence of the two ion species and the collisions between them. The greater number of protons passing through the shock is consistent with higher proton temperatures^31^. In contrast, panels b, c, e and f show the ion acceleration dissipation mechanism of CES clearly, with a double-layer structure and significant acceleration of protons.

**Figure 3:**
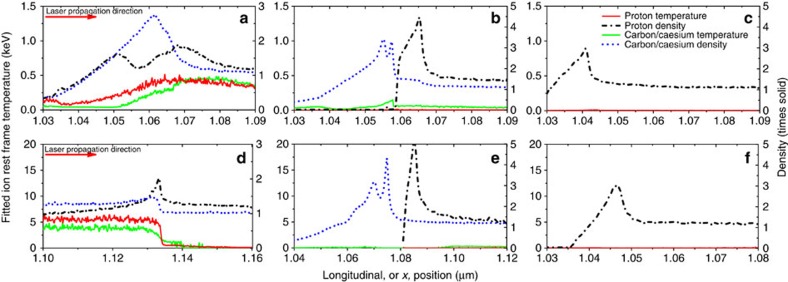
Temperature and density profiles centred on the maximum density point 40 fs into the PIC simulations. (**a**,**d**) All collisions enabled; (**b**,**e**) collisions between different ion species disabled; (**c**,**f**) all collisions enabled but the material is made up of protons only at the same density as the protons in **a**,**d**, respectively; (**a**–**c**) CH target, *I*=10^20^ W cm^−2^; (**d**–**f**) CsH target, *I*=5 × 10^20^ W cm^−2^. Red arrows show the laser propagation direction. The temperatures, fitted in the proton rest frame using a Maxwell–Boltzmann distribution, show a clear increase when collisions between different ion species are included as in **a**,**d**. The temperatures are the mean of the temperatures in the parallel and perpendicular directions.

Figure 3 shows the final temperatures and densities of the ions as a function of distance, centred around the point of maximum density. For the panels in which ultrafast collisional ion heating cannot operate, b, c, e and f, the ion temperatures are significantly lower. In scenarios b, c, e and f, there is only a relatively small temperature change across the shock front but the presence of collisions between ions of different species in scenario a and d changes this behaviour considerably.

**Figure 4:**
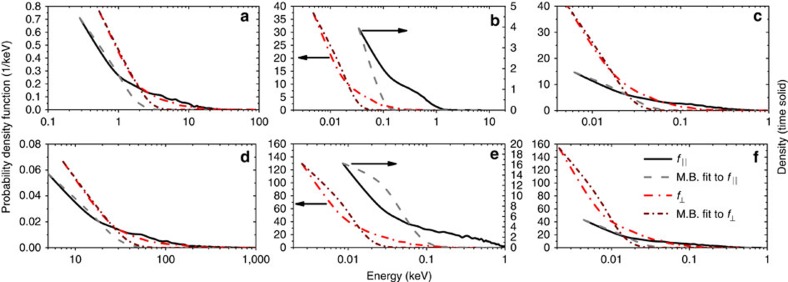
The ion rest frame distribution functions and Maxwell–Boltzmann (M.B.) fits to them in directions perpendicular and parallel to the laser pulse propagation direction 20 fs into the PIC simulations. These are taken at the point in the target with the highest density and provide an example of how Maxwell–Boltzmann distributions are fitted to the proton distribution functions in the rest frame of the protons to produce the temperatures shown in Figs 1, 3 and 5. Black arrows indicate the appropriate *y* axis scale for a particular distribution function and its fit. Solid and dash lines represent the distributions parallel to the shock; dash-dot lines the distributions perpendicular to it. (**a**,**d**) All collisions enabled; (**b**,**e**) collisions between different ion species disabled; (**c**,**f**) all collisions enabled but the material is made up of protons only at the same density as the protons in **a**,**d** respectively; (**a**–**c**) CH target, *I*=10^20^ W cm^−2^; (**d**–**f**) CsH target, *I*=5 × 10^20^ W cm^−2^.

The temperatures are calculated by fitting Maxwell–Boltzmann distributions to the ions' distribution functions in their rest frame. This ensures directed kinetic energy is not mistakenly accounted for as thermal energy. This is an important distinction, because some protons are still reflected from the shock in the scenario where ultrafast collisional ion heating takes place. Examples of the fits to the bulk distributions, which yield the reported temperatures, are shown in Fig. 4 for protons. The distribution functions remain anisotropic on time scales less than a few femtoseconds; thus, Maxwell–Boltzmann distributions are fitted separately in the parallel and perpendicular directions, with the quoted temperatures an average. In Fig. 4a,d, the fits are good, except for the high-energy tails that are in part due to reflection from the shock front and which play an unimportant role in ultrafast collisional ion heating. Ion–ion collisions allow the proton distribution to remain quasi-Maxwellian during the rapid heating. It is noteworthy that the large difference in the scale of the energy axes of the plots in panels b, c, e and f relative to panels a and d of Fig. 4 is caused by ultrafast collisional ion heating.

The rapid rate of ion heating is a key feature of this mechanism and is clearly shown in Fig. 1 for the two different materials. Figure 1 shows scenario a and d, in which collisions occur between all species (electron and ion) and there are two ion species in the target material.

**Figure 5:**
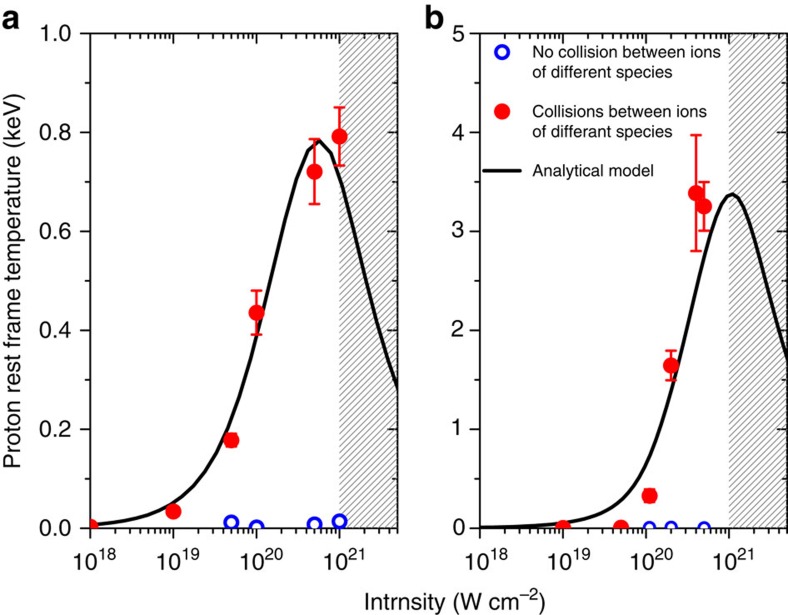
A comparison of the proton heating found in PIC simulations (red dots) and the heating predicted by the analytical model (black line). These are calculated 40 fs into the PIC simulations. Temperatures are measured at the highest density point in the target and the ten computational cells on either side of this point (roughly a skin depth *δ*_sk_). The error bars are given by the s.d. of the fitted temperature across these cells. The highest density point is always at, or above, solid density. Also shown are hollow blue dots, which show the heating achieved in simulations with collisions between different species of ion disabled. The patterned region denotes where a Maxwell–Boltzmann distribution does not provide a good fit for the proton distribution function and so the analytical model no longer applies. (**a**) CH target, (**b**) CsH target.

An analytical model of the ion heating is described in the Methods section. The results of this model for the proton temperature as a function of intensity are shown in Fig. 5. The fit to the model is fair for both materials, although superior for CH as shown in panel a. The model successfully explains the rate of increase in the final temperature with intensity and demonstrates that reaching these temperatures through ultrafast collisional ion heating is feasible. The much lower temperatures achieved in the particle in cell (PIC) simulations of scenario b and e, where collisions between ions of different species are disabled, are also shown, adding further evidence that the heating is predominantly due to ultrafast collisional ion heating. The model predicts an eventual reduction in temperature at the highest intensities, because the high relative velocities achieved by the ions results in them ceasing to be collisional (the basic Landau–Spitzer ion–ion collision frequency scales as 
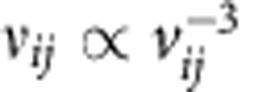
) and this suppresses the heating rate. However, it is collisions that keep the ions' distribution functions Maxwellian; thus, the model breaks down as the ions cease to be collisional, more protons are reflected by the shock and the proton distribution is no longer well approximated by a Maxwell–Boltzmann distribution. This is shown by the patterned region.

The energies of the protons were analysed. The amount of energy that went into thermal heating of protons due to ultrafast collisional ion heating was determined using the Maxwell–Boltzmann fits to the temperature. For CH, the peak proton thermal to kinetic energy ratio was 33%, representing 0.02% of the total laser energy at *I*=10^20^ W cm^−2^ at the end of the pulse in the scenario where ultrafast collisional ion heating acted. The ratio of thermal energy to kinetic energy was lower for all other intensities in the same scenario, being ∼1% for the intensities simulated. The thermal-to-laser energy ratio was similarly reduced at other intensities. For the two scenarios in which ultrafast collisional ion heating cannot act, the ratio of laser energy into thermal energy peaks at ∼10^−5^ % in both cases.

In CsH, the peak ratio of thermal energy to directed kinetic energy was 13% with *I*=2 × 10^20^ W cm^−2^. The peak ratio of thermal energy to laser energy was 0.005% compared with ∼10^−4^ % for scenarios b, c, e and f.

These laser energy conversion efficiencies are too small for this mechanism to be useful purely as a method of heating matter. However, the change relative to ignoring ultrafast collisional ion heating is an order of magnitude or more.

In these simulations, it is assumed that the laser has no pre-pulse. Experimental facilities have a range of laser pre-pulses, which may alter the target before the main pulse arrives, but the pre-pulse varies from shot to shot and between facilities; thus, the approximation of no pre-pulse is a useful simplification. Pre-pulses may create lower density pre-plasmas on the surface of targets. The presence of significant pre-plasma prevents CESs from reaching the solid density part of the target and reduces the heating rate due to ultrafast collisional ion heating because of its lower density. However, techniques such as plasma mirrors can vastly improve laser pre-pulses^1^. Alternatively, the longer pulses of up to 500 fs available on high-intensity laser facilities have enough energy for the main laser pulse to penetrate the pre-plasma^34^,^16^ and move the situation back to that of a CES being launched into solid density matter.

Hot electrons play an indirect role in CES as the amount of electron refluxing, determined by target thickness, influences the shock speed *v*_sh_ (ref. 20). For intensities that are strong enough to launch a shock, ultrafast collisional ion heating occurs and is significant, but the amount of heating depends on intensity given a particular target thickness; lower shock velocities are associated with thicker targets. The effect of substantially increasing target thickness would be to shift the heating curve shown in Fig. 5 to higher intensities. However, target thicknesses that are larger by an order of magnitude or more are required to cause a significant change in *v*_sh_/*c*; thus, the results reflect what is achievable for moderate thickness targets *L* where *L*≤5*λ*(*m*_*i*_/*m*_*e*_)^1/2^(*n*_cr_/*n*_*i*_)^1/2^/(*N*_electron cycles_)) where *N*_electron cycles_>1, the critical density is 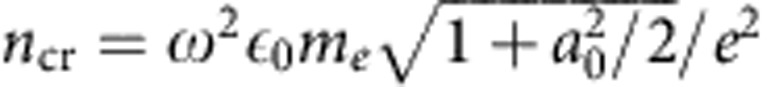 and the laser strength parameter is *a*_0_=*eE*_0_/*m*_*e*_*ωc*.

The PIC simulations performed include other heating mechanisms such as electron–ion collisions, electron–ion and ion–ion collisionless interactions such as two-stream instabilities and the stopping of fast ions reflected from the shock front heating the plasma upstream of the shock. Ion interactions with hot electrons and cold electron return currents are present in all simulations, but there is no significant heating in scenarios b, c, e and f in which collisions between ions of different species are disabled. Collisionless heating is included in all scenarios but is similarly unimportant. Fast ion stopping has a time scale of 
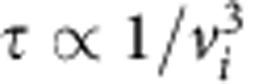
 for ion *i* and it is estimated^19^ that a target with incident radiation of *I*=10^20^ W cm^−2^ produces a beam of reflected ions with a slow down time of 1 ps and a deposition length around 1 μm. This is a longer time than is relevant to the simulations presented and the deposition length scale extends far beyond the shock width. There is very little ion heating upstream of the shock observed in the figures. As it is protons that are reflected from the shock, this effect would also be apparent in panels c and f of Figs 2, 3, 4 if it were significant. Therefore, the only significant change to final temperatures occurs when collisions between ions of different species are included and ultrafast collisional ion heating can act.

As the heated volume is very small, the practical uses of this heating mechanism may be few in number. Most of the target (ahead of the shock is) remains cold, with a proton beam passing through it, or is behind the shock and is heated but is, on longer time scales, also being ablated from the front surface by the direct interaction of the laser. However, one interesting implication of ultrafast collisional ion heating is that ion acceleration schemes relying on laser–solid interactions may produce less high-quality beams^35^ than previously anticipated as measured by the metric of transverse beam emittance, which is the average spread in *P*_*y*_−*y* space where *y* is the spatial dimension transverse to the beam. Some of these schemes rely on accelerating ions from small amounts of solid density material, meaning the heating effect we describe could be important for their dynamics.

## Discussion

We have demonstrated a new heating mechanism in which radiation pressure-driven electrostatic shocks can produce proton temperatures of the order of keV at solid densities on time scales of the order of tens of femtoseconds. The heating mechanism relies on ion–ion collisions between different species of ion, which are not typically assumed to be important in dissipating CESs and is unusual in that ions are not heated directly by their interactions with electrons. The modest volume heated by this mechanism means that it may have few practical applications (unless the effect could somehow be enhanced), although it could have implications for ion acceleration schemes. The conditions required for ultrafast collisional ion heating are within the capabilities of current laser facilities. If this heating mechanism is demonstrated experimentally, the rate of ion heating will be, to our knowledge, the fastest achieved for a significant number (>10^12^) of particles.

## Methods

### PIC simulations

Simulations were perfomed with the freely available relativistic epoch PIC code^36^, which includes an algorithm for performing collisions between all particles^37^. All particles, electron and ion, collide with all other particles unless explicitly stated as in panels b and e of Figs 2, 3, 4 and temperatures represented by hollow circles in Fig. 5. The laser pulses have wavelength *λ*=800 nm enter the simulation domain at *x*=0 and have a Gaussian temporal shape of full width half maximum 15 fs, with peak intensities quoted. The laser pulse is linearly polarized. The one-dimensional simulation domain is 1.5 μm thick and longitudinal positions are measured relative to the pulse entry point at *x*=0 μm. Targets are 0.3 μm thick, far greater than the skin depth *δ*_sk_=*c*/*ω*_pe_, and are positioned between *x*=1 μm and *x*=1.3 μm. The ions are initialized cold, whereas *T*_*e*_(*t*=0)=100 eV, to satisfy the constraints of the collision algorithm. Two targets are simulated: plastic (equimolar carbon and protons, CH) and CsH. In all simulations, the simulation domain is resolved by a grid of 10,000 cells with 1,200 particles per cell (400 per species for both electrons and ions). The plastic initial density is *ρ*=1.04 g cm^−3^ and the skin depth of *δ*_sk_=8.8 nm is resolved. Ions in CH targets are assumed to be fully ionized. CsH targets were simulated at the solid density of *ρ*=3.42 g cm^−3^ and the skin depth of *δ*_sk_=8.0 nm is resolved. Cs is not fully ionized and we assume Z*=27 corresponding to over-the-barrier ionization of atomic energy levels down to principal quantum number *n*=4. This is denoted throughout the text as *Z*_*i*_ or *Z*_*j*_ for notational convenience. The thermal energy to laser energy ratios quoted for the two scenarios, 2 and 3, where ultrafast collisional ion heating cannot act, are determined from two-thirds of the median energy in the protons' rest frame. The Debye length, *λ*_D_, is larger than the inter-ion particle radius for all simulations.

### Analytical model of heating

The analytical model of the heating effect, shown in Fig. 5, is derived from the Boltzmann equation. The model is based on two different populations of ions being differentially accelerated by the electric field, *E*_*x*_, associated with the shock. The friction between the two species of ions is the cause of their heating. It is assumed that the accelerated ions of two distinct ion species *i* and *j* each have Maxwell–Boltzmann distributions moving at velocities *v*_*i*_ and *v*_*j*_, respectively, in the laboratory frame and have relative velocity *v*_*ij*_=*v*_*i*_−*v*_*j*_. Differential equations may then be expressed for the rate of change of temperatures with respect to the distance, 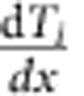 and 
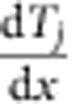
, and the rate of change of bulk velocity of the ion distributions with respect to the distance, 
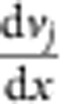
 and 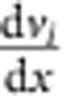. These are


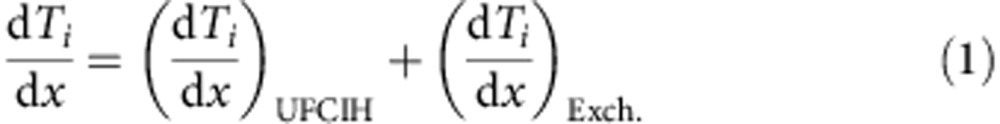



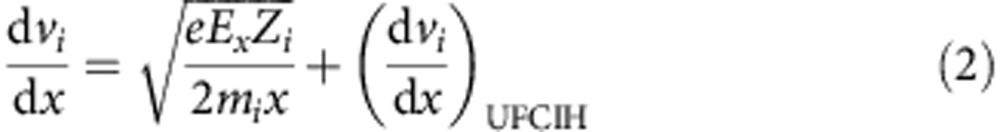


and similarly for *j*. The first term on the right-hand side of equation (2) simply comes about due to the accelerating field of the shock, *E*_*x*_. ‘UFCIH' represents the ultrafast collisional ion heating term, whereas ‘Exch.' represents an ion–ion temperature equilibration term. These terms are given by^38^,^39^


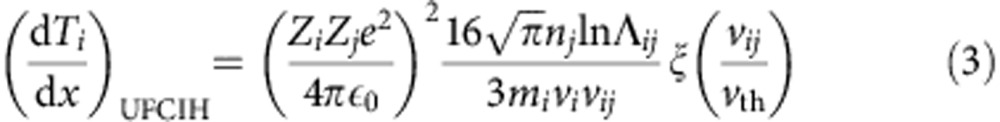



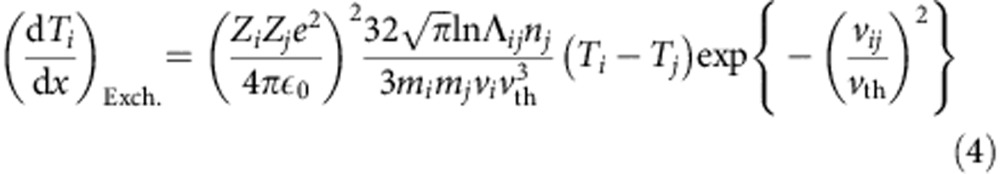



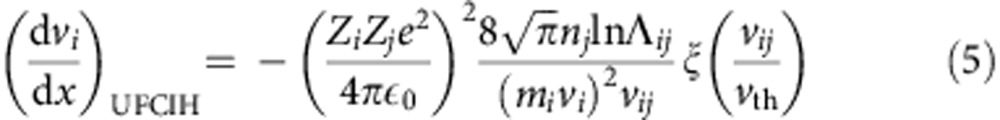


where


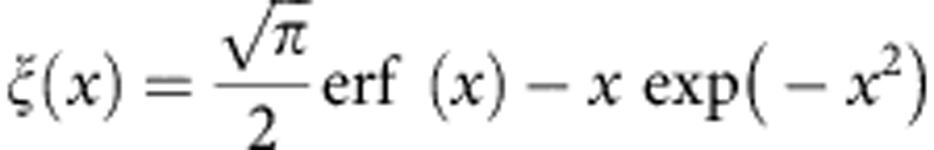


erf(*x*) is the error function and 
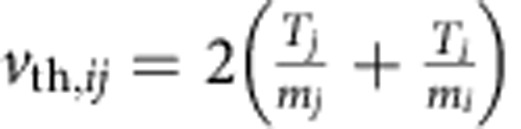
.

The equations are solved by the classical Runge–Kutta method. The only variable input to the model for each material is the incident laser intensity. Other variables, which do not change as a function of intensity, are the Coulomb logarithm (set to lnΛ=4 in the analytical model), shock width *L* and the ion densities *n*_*i*_, *n*_*j*_. The shock widths and densities used in the analytical model are representative values taken from simulations: *L*=15 nm and *n*_*i*_=5*n*_*i*_(*t*=0) for CH, *L*=24 nm and *n*_*i*_=5*n*_*i*_(*t*=0) for CsH. CES have widths on the order of the Debye length, *L*=(4–10)*λ*_D_ (refs 18, 27). The electric field of the shock, *E*_*x*_, is given as a function of incident intensity by the electrostatic shock efficiency relation^19^ so that 
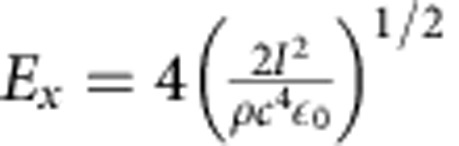
 where *I* is the incident laser intensity.

## Additional information

**How to cite this article:** Turrell, A. E. *et al.* Ultrafast collisional ion heating by electrostatic shocks. *Nat. Commun.* 6:8905 doi: 10.1038/ncomms9905 (2015).
